# Organic amendment additions to cadmium-contaminated soils for phytostabilization of three bioenergy crops

**DOI:** 10.1038/s41598-022-17385-8

**Published:** 2022-07-29

**Authors:** Puntaree Taeprayoon, Kunaporn Homyog, Weeradej Meeinkuirt

**Affiliations:** 1 Agricultural and Environmental Utilization Research Unit, Nakhonsawan Campus, Mahidol University, Nakhonsawan, 60130 Thailand; 2 The Center for Veterinary Diagnosis, Faculty of Veterinary Science, Mahidol University, Nakhon Pathom, 73170 Thailand; 3 Water and Soil Environmental Research Unit, Nakhonsawan Campus, Mahidol University, Nakhonsawan, 60130 Thailand

**Keywords:** Plant sciences, Environmental sciences, Natural hazards

## Abstract

The effects of organic amendments on the phytoremediation of cadmium (Cd) in acacia (*Acacia mangium*), jatropha (*Jatropha curcas*), and cassava (*Manihot esculenta*) were investigated. The bone meal/bat manure and leonardite/bat manure amendments resulted in better growth performance in both acacia and cassava (growth rate in dry biomass; GRDB 24.2 and 22.2, respectively), while bone meal (GRDB 17.2) was best for jatropha. The lower root/shoot ratio values of jatropha and acacia suggest that these species were better suited than cassava on Cd-contaminated soil. Cassava experienced toxicity symptoms after harvest (3 months). Acacia root accumulated somewhat greater Cd concentrations (up to 5.1 mg kg^−1^) than cassava and jatropha roots (2.2–3.9 and 2.7–4.1 mg kg^−1^, respectively). The bone meal and chicken manure (BMCM) treatment for jatropha had the highest bioconcentration factor for root (1.3) and the lowest translocation factor (0.7). Despite the fact that this treatment had substantial Cd concentrations in the soil (3.1 mg kg^−1^), low Cd accumulation value (3.2 mg kg^−1^) and the lowest Cd uptake value (127.8 mg plant^−1^) were observed, clearly indicating that this amendment reduced Cd bioavailability. When growth performance of the study plants is considered, jatropha and acacia may be suitable for phytomanagement of Cd-contaminated soil.

## Introduction

The smelting and mining of zinc (Zn) ore in the Padaeng Zn mine in Mae Sot District, Tak Province, Thailand has resulted in the inadvertent release of cadmium (Cd) to a predominantly agricultural ecosystem. Soils contaminated with high concentrations of Cd seriously impact crop development and growth, and result in decreased crop yields^1^. Cadmium is among the most toxic metals to humans with sublethal behavioral, carcinogenic, and mutagenic effects, even at low concentrations. Furthermore, it is recognized that the potential for bioaccumulation and biomagnification of Cd in food chains contributes to its long-distance transport, which suggests that Mae Sot’s population (~ 120,000) is at elevated risk of Cd exposure as a result of consumption of Cd-affected crops^2^.

Phytoremediation is a recently developed and viable alternative technology for ameliorating inorganic and organic toxic pollution in soil, surface water, and groundwater using green plants^3^. Phytoremediation can potentially be enhanced by introducing soil amendments that improve the growth and remediation capabilities of selected plants. Organic amendments are suitable in many heavily polluted environments as they promote both plant growth and phytoremediation potential. Such materials are readily available, low in cost, and simple to apply. In Cd-contaminated soil, pig and cattle manure, leonardite, and other organic additions help stabilize Cd, reducing both uptake by plants and translocation in plant tissues^4,5^. So-called phytomanagement, which combines phytostabilization and crop production with the use of organic amendments, is a highly effective strategy for treating metal-enriched soil. Non-edible plant products having low Cd concentrations can be used for biofuel, fiber, wood, and possibly animal feed^6^.

Several studies have determined that dicotyledonous plants are better suited for phytostabilization than monocots are, as they tend to produce greater biomass and have a longer lifespan^7^. Some dicotyledonous crop plants, such as acacia (*Acacia mangium* Willd.) are capable of accumulating nickel (Ni) and chromium (Cr), while jatropha (*Jatropha curcas* L.) accumulates copper (Cu), Zn, Cd, and lead (Pb). Both exhibit substantially higher heavy metal accumulation in roots compared with shoots and are termed excluders^8^. Cassava (*Manihot esculenta* Crantz), on the other hand, is a hyperaccumulator and is employed in mercury (Hg) and gold (Au) phytoextraction because it accumulates significant quantities of both metals in shoots^9^.

The purpose of the reported study was to determine suitable organic amendments for enhancing phytostabilization of Cd by three bioenergy crops [acacia (*A. mangium*), jatropha (*J. curcas*), and cassava (*M. esculenta*)] grown in Cd-contaminated soil under greenhouse conditions. The bioenergy crop considered superior with organic amendment application, as indicated by survival rate, growth performance, and Cd accumulation in roots, is recommended for phytomanagement as it can be cultivated in contaminated soil and used for energy purposes.

## Materials and methods

### Plants

The crop plants used in this study were acacia (*A. mangium*), jatropha (*J. curcas*), and cassava (*M. esculenta*). Acacia seedlings were purchased from farms in Nakhon Sawan Province. Cassava stalks were designated as Rayong No. 13, which is promoted by the Thailand Department of Agriculture and purchased from farms in Nakhon Sawan Province, whereas jatropha seedlings were obtained from a farm in Phitsanulok Province, Thailand. Jatropha and cassava were acclimatized by cultivation for 1 month in uncontaminated commercial soil placed in plastic containers, while acacia was acclimatized for 7 days prior to start of the experiments. All study plants were placed on benches in a greenhouse under controlled conditions (27 to 29 °C; 63 to 75% relative humidity; 10,253 to 15,631 lx light intensity; 12/12-h photoperiod) before being transferred to pot systems. Uniform and healthy plants were selected for pot experiments.

### Pot experimental design

All methods used to determine phytoremediation potential of the study plants (e.g., cultivation methods, heavy metal determination) were carried out according to local guidelines ensuring ethically conducted research. Non-contaminated soil material was purchased from an agricultural farm in Nakhon Sawan Province. Soil material was thoroughly mixed and then ground with an agate mortar and pestle before being passed through a 2-mm sieve. Two and one-half kg of soil was mixed with an amendment, placed into plastic pots (10 in diameter, 7.5 in height), and allowed to equilibrate for 1 month. The ratio of amendment to soil was 1:6.7. Organic amendments were applied to soil as follows: 375 g leonardite (SL), 375 g bone meal (SBM), 187.5 g leonardite/187.5 g bone meal (SLBM), 250 g leonardite/125 g vermicompost (SLVC), 250 g leonardite/125 g chicken manure (LCM), 250 g leonardite/125 g bat manure (LBM), 250 g bone meal/125 g vermicompost (BMVC), 250 g bone meal/125 g chicken manure (BMCM) and 250 g bone meal/125 g bat manure (BMBM).

One replicate was represented by a single pot with a single plant. Each treatment had four replicates. Pots were placed in a randomized complete block design (RCBD) on greenhouse benches. Plants were treated daily with 255.4 ± 127.5 mL Cd-enriched water throughout the experiment. Cadmium nitrate [Cd(NO_3_)_2_; Merck^®^, Germany] was the source of Cd. Solution was added at the rate of 0.064 mg Cd L^−1^. To maintain proper nutrient levels in soil, each pot was supplemented with 6 g Osmocote at 1.5 months.

### Soil analysis

Soil texture, organic matter (OM) content, cation exchange capacity (CEC), total nitrogen (total N), extractable phosphorus (Ext. P), extractable potassium (Ext. K), extractable calcium (Ext. Ca) and extractable magnesium (Ext. Mg) were measured using APHA, AWWA, and WEF procedures^10^. A pH meter (Accumet AP115) and EC meter (Hanna instruments; HI 993310) were used to measure pH and electrical conductivity (EC) of the soil in a 1:5 soil/water suspension. Physicochemical properties were also measured in a soil sample without amendment (termed control). Maintaining water saturation in soils from 70 to 80% was carried out using the method of Blaylock et al.^11^.

### Cadmium determination in plants and soil

After 3 months, plants were carefully removed from pots, washed thoroughly with tap water to remove excess soil, and rinsed with distilled water and DI water, sequentially. Plants were divided into leaves, stems and roots, packed in paper bags and oven-dried for 5 days at 70 °C. Plant tissue was weighed, ground with a mortar and pestle (IKA; A11 basic) and sieved through a 2-mm nylon mesh screen to obtain a fine powder. In a microwave digester (ETHOS One; Milestone Inc.), 0.5 g dry weight (DW) of ground tissue was digested with concentrated 70% nitric acid (HNO_3_) and 37% hydrochloric acid (HCl), while 0.5 g of soil sample was digested with concentrated 70% HNO_3_ and 30% hydrogen peroxide (H_2_O_2_). The digested samples were filtered using Whatman No. 42 filter paper and then diluted to 25 mL with 1% HNO_3_ in DI water. All reagents employed in this study were TraceMetal grade from Merck, Germany. Depending on Cd concentration, FAAS or GF-AAS were used to analyze plant and soil digests. A blank method, and soil and plant standard reference materials (NIST SRM 2710a Montana soil and NIST SRM 1515 apple leaves, respectively) were used to determine accuracy of sample data. Percent recovery rates for plant and soil samples were 91.4 to 109.3% and 94.2 to 109.2%, respectively. Relative standard deviation (RSD) values of plant and soil samples varied from 1.3 to 3.3% and 1.6 to 4.3%, respectively. RSD values of the samples were less than 5%, indicating precision of the method.

### Data collection and statistical analysis

The bioconcentration factor of root (BCFR) was calculated by Cd concentration of root/ext. Cd in soil^12^. Translocation factor (TF) was calculated by Cd concentration in plant shoots/Cd concentration in roots^12^. Cadmium uptake was calculated by Cd concentration in whole plant tissue × plant dry biomass^13^. Growth rate in dry biomass (GRDB) was calculated by plant dry biomass after harvest – (plant dry biomass before planting/number of months of plant growth)^5^. Root:shoot (R/S ratio) was calculated by total dry biomass of root/total dry biomass of shoots^14^.

Data was statistically analyzed using SPSS, Version 18.0. The statistical difference among treatments for each plant species and among plant species in each treatment was analyzed using one-way analysis of variance (one-way ANOVA), followed by least significant difference (LSD) *post-hoc* comparisons at the 95% confidence level (*p* < 0.05). All data were expressed as mean ± standard deviation (S.D.).

## Results and discussion

### Physicochemical properties of amended soils

The texture of commercial soil (control soil) was loam; however, the texture of certain amended soils (SL, SLBM, SLVC, LCM, and LBM) changed to clay loam (Table 1). Loam texture has good physical properties and typically contains adequate levels of nutrients, organic matter and moisture, making it ideal for horticultural purposes. Loam is the most common soil texture in the Cd- and Zn-contaminated soils in Mae Sot District, Tak Province, Thailand^1^. Organic amendments raised the pH of some amended soils, *e.g.*, SL, SBM, SLBM, LBM, BMVC, BMCM, and BMBM to a range of 6.0–7.3, while SLVC- and LCM-amended soils resulted in slight reductions in pH (5.5 and 5.8, respectively). Leonardite used in combination with SLVC, LCM, and LBM has the potential to lower soil pH; therefore, leonardite is usually mixed with other amendments to achieve the desired soil pH for plant cultivation^15^. Electrical conductivity values in SL, SLVC, LCM and BMCM were relatively high (2.1 to 2.5 dS m^−1^), exceeding the regulatory limit of 2 dS m^−1^. This level of salinity could adversely affect crop growth and microbial activity^16^. Organic amendments can also raise EC to excessively high levels, necessitating the evaluation of any symptoms likely to indicate damage to the study plants^17^. Addition of the organic amendments resulted in increases in OM content over the control soil by a factor of 1.3 to 1.5. The CEC values of some amended soils, such as SBM, BMVC, BMCM, and BMBM decreased slightly, which could be result of a dilution effect of the amendments^5^. The amendments may also enhance pH buffering capacity, percent base saturation, soil enzymatic activity and nutrient availability (e.g., available P and exchangeable NH_4_^+^)^18^. When compared with a commercial soil in a related study, the control soil in this study had higher concentrations of essential nutrients (N, P, K); however, it had lower concentrations of Ca and Mg^14^. Organic amendments increased essential nutrient concentrations such as total N, Ext. P, Ext. Ca, and Ext. Mg; however, Ext. K content was somewhat lower in the amended soils, with the exception of the BMCM treatment (3960 mg kg^−1^) which was higher than the control treatment by a factor of 1.4 (Table 1).

**Table 1:** Physicochemical properties of the amended soils.

	Parameter	Units	Treatment									
			Control	SL	SBM	SLBM	SLVC	LCM	LBM	BMVC	BMCM	BMBM
	Texture		Loam	Clay loam	Loam	Clay loam	Clay loam	Clay loam	Clay loam	Loam	Loam	Loam
	Sand	%	44.8	42.4	43.4	42.4	40.2	39.6	42.2	44.0	48.2	48.2
	Silt	%	37.6	28.2	31.2	28.2	29.2	32.2	28.2	30.2	28.0	28.0
	Clay	%	17.6	29.4	25.4	29.4	30.6	28.2	29.6	25.8	23.8	23.8
	pH		5.9	6.6	7.3	6.9	5.5	5.8	6.0	6.8	6.9	7.3
	EC	dS m^−1^	1.5	2.2	1.8	1.9	2.1	2.5	1.8	2.0	2.5	1.7
	OM	%	6.8	9.4	9.0	8.6	9.9	9.8	8.9	9.6	8.5	8.5
	CEC	cmol kg^−1^	13.4	21.5	11.8	15.6	22.1	20.2	17.0	12.9	11.9	10.8
	Total N	%	0.34	0.47	0.45	0.43	0.49	0.49	0.44	0.48	0.43	0.42
	Ext. P	mg kg^−1^	467	441	5928	4375	866	715	2302	5676	6363	5975
	Ext. K	mg kg^−1^	2749	2586	2478	2391	2305	2723	2085	2530	3960	2679
	Ext. Ca	mg kg^−1^	1375	3948	3682	4447	2832	2244	2874	3640	2928	3768
	Ext. Mg	mg kg^−1^	463	561	511	547	708	702	514	667	690	532
**Cd concentrations in soil prior to planting**												
	Total Cd	mg kg^−1^	BDL	BDL	BDL	BDL	BDL	BDL	BDL	BDL	BDL	BDL
	Ext. Cd	mg kg^−1^	BDL	BDL	BDL	BDL	BDL	BDL	BDL	BDL	BDL	BDL
**Cd concentrations in soil following plant harvest**												
*J. curcas*	Total Cd	mg kg^−1^	–	2.7 ± 1.2bA	2.9 ± 1.2bA	2.5 ± 1.3 bA	2.0 ± 0.9 bA	2.3 ± 1.1abA	3.1 ± 1.1bA	3.7 ± 1.3bA	3.1 ± 1.4aA	2.6 ± 1.2abA
	Ext. Cd	mg kg^−1^	–	0.7 ± 0.2bA	0.7 ± 0.2bA	0.5 ± 0.2 bA	0.4 ± 0.2 bA	0.7 ± 0.2abA	0.4 ± 0.2bA	0.7 ± 0.2bA	0.7 ± 0.2aA	0.4 ± 0.2abA
*M. esculenta*	Total Cd	mg kg^−1^	–	0.6 ± 0.3bcB	1.0 ± 0.3aB	0.6 ± 0.3abcB	0.4 ± 0.2cB	0.4 ± 0.2cB	0.5 ± 0.0cB	0.6 ± 0.4bcB	0.4 ± 0.cB	0.9 ± 0.6abB
	Ext. Cd	mg kg^−1^	–	0.5 ± 0.1bcdB	0.6 ± 0.1aA	0.5 ± 0.1bcA	0.5 ± 0.1bcdA	0.4 ± 0.1deB	0.3 ± 0.1eA	0.4 ± 0.1cdeB	0.5 ± 0.1abB	0.4 ± 0.1bcdA
*A. mangium*	Total Cd	mg kg^−1^	–	1.5 ± 0.9 bB	2.4 ± 0.7 bA	2.5 ± 1.3 bA	2.3 ± 0.3 bcA	2.8 ± 0.6abA	3.2 ± 0.1abA	3.5 ± 0.3aA	2.8 ± 0.3abA	2.9 ± 0.1abA
	Ext. Cd	mg kg^−1^	–	0.6 ± 0.1 aAB	0.6 ± 0.0 aA	0.5 ± 0.0 bcA	0.4 ± 0.1 cA	0.6 ± 0.1abA	0.4 ± 0.1cA	0.5 ± 0.1abcAB	0.6 ± 0.1aAB	0.5 ± 0.1cA

*BDL* below detection limits*, CEC* cation exchange capacity*, EC* electrical conductivity*, Ext.* extractable*, OM* organic matter*, Control* commercial soil*, SL* soil + leonardite*, SBM* soil + bone meal*, SLBM* soil + leonardite + bone meal*, SLVC* soil + leonardite + earthworm manure*, LCM* soil + leonardite + chicken manure*, LBM* soil + leonardite + bat manure*, BMVC* soil + bone meal + earthworm manure*, BMCM* soil + bone meal + chicken manure*, BMBM* soil + bone meal + bat manure.

Values followed by the same letter are not significantly different; lower-case letters show the differences in Cd concentrations in the soil within the same plant species (LSD, *p* < 0.05); capital letters show the differences in total (or Ext.) Cd concentrations in the soils among plant species (LSD, *p* < 0.05).

Before planting, total Cd and Ext. Cd concentrations in all soil samples were below detection limits (Table 1), but values increased somewhat following the plant harvest. Total Cd concentrations were greater than Ext. Cd concentrations. Fertilizers used in paddy fields typically are phosphate-bearing rock material; this material, as well as local irrigation water delivered to paddy fields, are key sources of the elevated Cd concentrations in agricultural soil in the Mae Sot region^19^. The Cd present in soil after 3 months is attributed to the addition of Cd solution and different uptake capabilities of the study plants. When comparing the amended soils, total and Ext. Cd contents for individual plant treatments exhibited narrow variations. Total Cd concentrations in all amended soils for jatropha, cassava, and acacia were 2 to 3.7 mg kg^−1^, 0.4 to 1 mg kg^−1^, and 1.5 to 3.5 mg kg^−1^, respectively, whereas Ext. Cd concentrations for jatropha, cassava, and acacia were 0.4 to 0.7 mg kg^−1^, 0.3 to 0.6 mg kg^−1^, and 0.4 to 0.6 mg kg^−1^, respectively. The BMVC and BMCM treatments for *J. curcus* showed substantial soil Cd concentrations (total Cd, 3.7 and 3.1 mg kg^−1^, respectively and Ext. Cd, 0.7 mg kg^−1^).

### Effects of amendments on plant growth

The jatropha and acacia thrived in Cd-treated soils for 3 months, with no visible phytotoxic effects in any of plants. Cassava also had a 100% survival rate, but showed symptoms of stress including deformed leaves and yellowing leaves in almost all treatments. Cassava cultivated in the SL treatment showed no symptoms of stress. Of the three crop plants surveyed, only cassava is cultivated in Cd- and Zn co-contaminated agricultural areas of the Mae Sot District, Tak Province.

**Figure 1:**
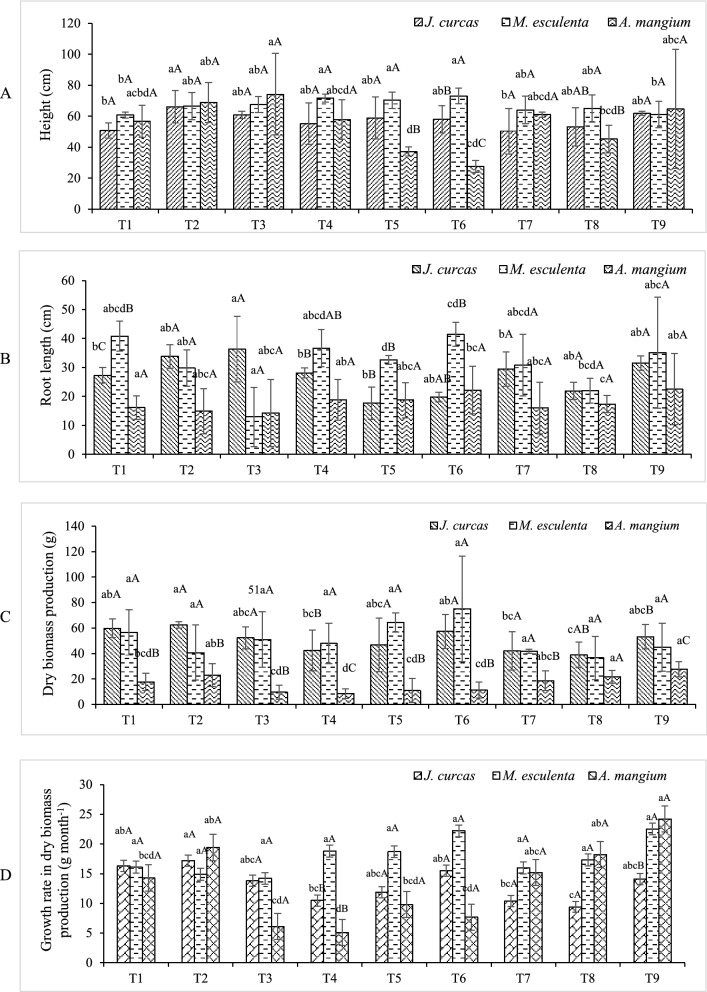
Growth performance of study plants and growth rate in dry biomass production, for 3 months (*n* = 4). Values followed by the same letter are not significantly differ; lower case letters show the difference in growth performance among treatments within the same plant species (LSD, *p* < 0.05; Fig. 2A–D); capital letters show the difference in growth performance among plant species within the same treatment (LSD: *p* < 0.05; Fig. 2D). T1 = SL, T2 = SBM, T3 = SLBM, T4 = SLVC, T5 = LCM, T6 = LBM, T7 = BMVC, T8 = BMCM, T9 = BMBM.

The amendments imparted positive effects on plant growth (height, root length, dry biomass production). Furthermore, all study plants in the amended treatments had considerably improved plant height and root length, as well as enhanced dry biomass production, from planting to harvesting periods (Fig. 1 and Table S1).

Except for jatropha, the GRDB values of the study plants revealed similar trends to plant growth performance, with GRDB values in month 3 slightly lower than month 1 (Table S1). After harvest, the BMBM treatment resulted in the best growth performance in acacia (dry biomass 27.7 g plant^−1^, GRDB 24.2), followed by the LBM treatment for cassava (dry biomass 75.1 g plant^−1^, GRDB 22.2). Height had the greatest influence on the plant dry mass (Fig. 1A, C). After harvest, GRDB values for the study plants were ranked in the order: cassava > acacia > jatropha (Fig. 1D).

Bone meal and bat manure are widely used as a P source in commercial fertilizer because of their high P content (7 to 12% and 1 to 9%, respectively). Bone meal has an NPK average content of 3-15-0, making it a rich source of P for plants^20^. Bat manure, commonly known as ‘guano,’ is an important organic source of N and P that also contains minerals such as struvite and magnesium, making it useful as a fertilizer material; however, the nutrient composition of guano varies depending on the diet of bats^20^. Leonardite is an end-product of lignite coal and mine pits at Mae Moh mine in Lampang Province. Leonardite has a high total N concentration (0.6%) but low Cd and Zn contents (0.7 mg Cd kg^−1^ and 40 mg Zn kg^−1^, respectively)^1^. It has been employed as an organic amendment to promote crop growth, particularly commercial Thai rice cultivars, thus indicating its effectiveness as a soil amendment^5^. According to acacia and cassava in this study, the treatment that received both bone meal and bat manure supplements had 1–1.1 times higher dry biomass after harvest than the treatment that just received bone meal. On the other hand, jatropha grew well in bone meal alone, as evidenced by the SBM treatment producing the highest biomass and GRDB compared with the other treatments (*p* < 0.05; 62.5 g plant^−1^, GRDB 17.2).

The LBM, SLVC, LCM, BMVC, and BMCM treatments exhibited lower growth performance, particularly in jatropha and acacia, providing further evidence that a specialized soil amendment such as bat, bone meal/or leonardite alone, is more beneficial. The combination of chicken manure or vermicompost with leonardite or bone meal was unable to support the growth of the study plants (Fig. 1 and Table S1). In Thailand's agricultural areas, vermicompost and chicken manure are important soil amendments. Both were reported to be successful in replacing chemical fertilizers and stimulating plant growth when combined with other organic materials, *e.g*., rice husk charcoal^21^. The decreased growth performance observed in the study plants grown with these amendments might be due to insufficient levels of the organic amendment; other studies have demonstrated that high concentrations of chicken manure alone promote plant growth performance as evidenced by significant impacts on plant height, total leaf count, stem diameter and harvested wet weight. It has also been observed that large additions of chicken manure and mushrooms can reduce heavy metal phytotoxicity^22^. Furthermore, the growth performance of cassava did not differ significantly across treatments (*p* > 0.05). This is a strong indication that the combination of chicken manure or vermicompost with leonardite or bone meal as soil organic amendments is a suitable alternative amendment for cassava.

**Figure 2:**
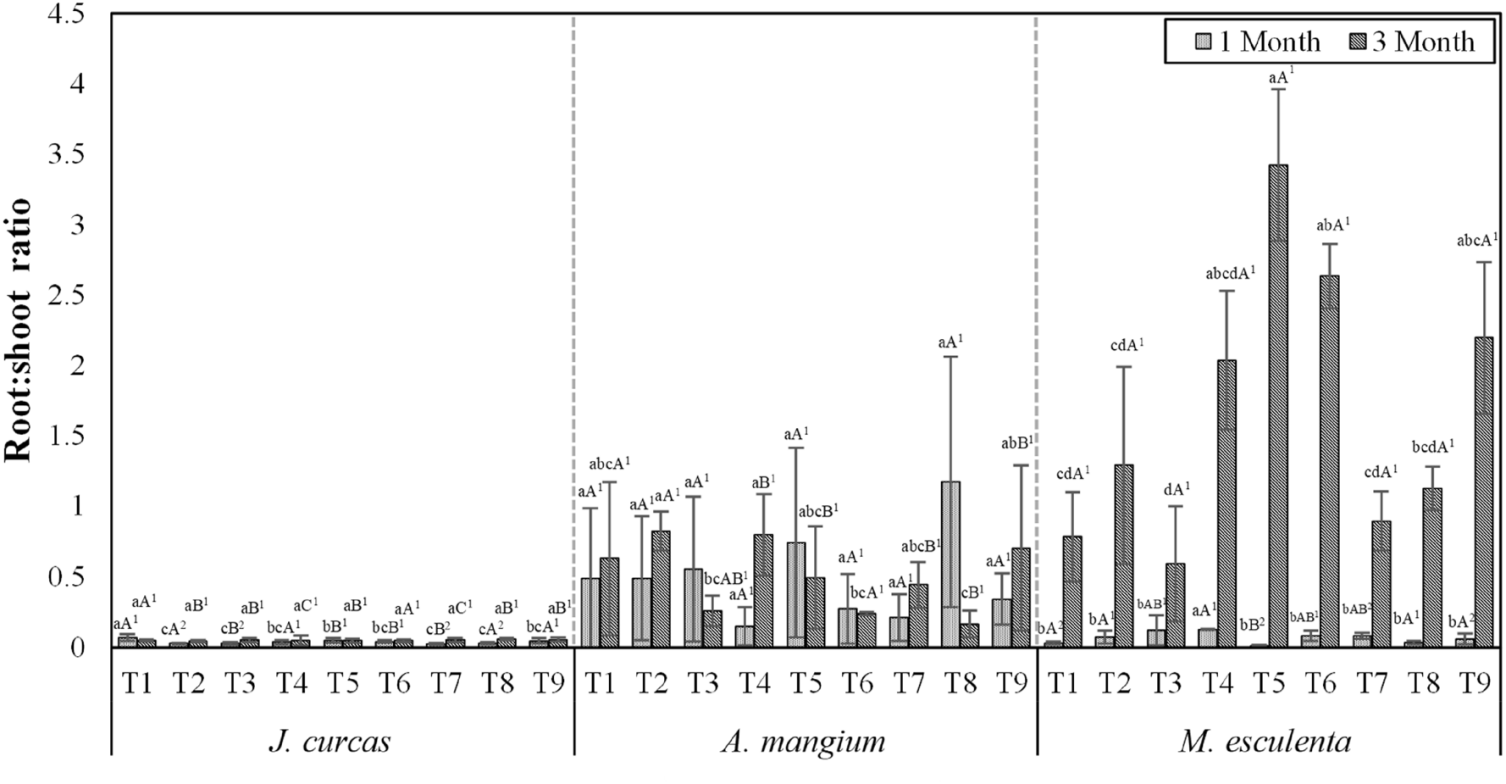
Root/shoot ratio of Cd in study plants. Values followed by the same letter are not significantly different; lower-case letters show the differences in R/S ratios among treatments within the same plant species and growth period (LSD: *p* < 0.05); capital letters show the differences in R/S ratios among plant species within the same treatment (LSD: *p* < 0.05); numbers indicate the differences in R/S ratios between growth periods within the same plant species and treatment. T1 = SL, T2 = SBM, T3 = SLBM, T4 = SLVC, T5 = LCM, T6 = LBM, T7 = BMVC, T8 = BMCM, T9 = BMBM.

Jatropha and acacia had lower R/S ratios than cassava (Fig. 2). The ascending order of R/S ratios of the study plants were: jatropha (0.04 to 0.06) < acacia (0.16 to 0.8) < cassava (0.59 to 3). According to Saengwilai et al.^5^, a high R/S ratio value indicates Cd toxicity to plants, and thus increased Cd concentration in plant media is considered harmful to the study plants. The R/S ratio values of jatropha and acacia in this study imply that these plants were healthier than cassava cultivated on Cd-treated soils. Substantial R/S ratio values were observed in all treatments for cassava, particularly in LCM and LBM treatments (3 and 2.63, respectively), which might be linked to increased plant stress.

### Cadmium concentrations in plant tissue

**Figure 3:**
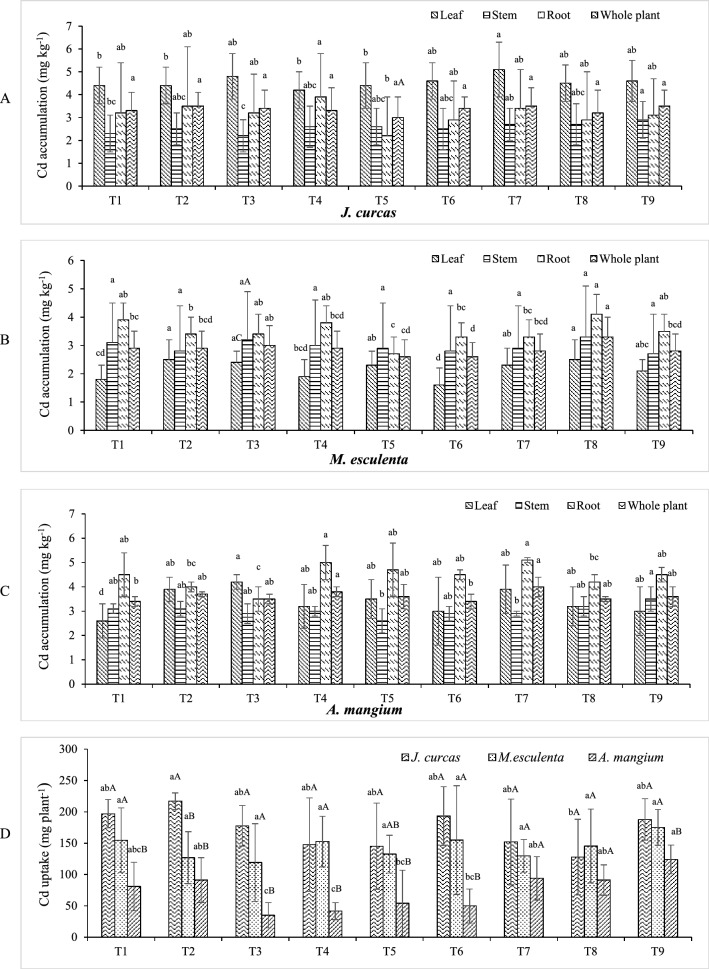
Cd accumulation and uptake among study plants, for 3 months (*n* = 4). Values followed by the same letter are not significantly different; lower-case letters indicate the difference of values in Cd uptake among treatments within the same plant species (LSD, *p* < 0.05); capital letters indicate the difference of values in Cd uptake among plant species within the same treatment (LSD: *p* < 0.05). T1 = SL, T2 = SBM, T3 = SLBM, T4 = SLVC, T5 = LCM, T6 = LBM, T7 = BMVC, T8 = BMCM, T9 = BMBM.

The amended soil treatments revealed a range of Cd accumulation levels and uptake depending on plant species (Fig. 3 and Table S2). Moreover, after 1 month of growth the study plants accumulated Cd primarily in roots, with average values of 2.4 to 6.2 mg kg^−1^, 4 to 15 mg kg^−1^, and 3.3 to 4 mg kg^−1^ for jatropha, cassava, and acacia, respectively. The LCM treatment resulted in maximum Cd accumulation (cassava, 15 mg kg^−1^), whereas the SLBM treatment resulted in the lowest value (acacia, 3.3 mg kg^−1^).

Following harvest, Cd accumulation in plants is generally ranked: leaves > roots > stems for jatropha and roots > stems ≈ leaves for cassava and acacia, respectively (Fig. 3A–C). Cadmium concentrations were higher in acacia roots across all treatments (3.5 to 5.1 mg kg^−1^), whereas cassava and jatropha had lower accumulation with narrower ranges (2.7 to 4.1 mg kg^−1^ and 2.2 to 3.9 mg kg^−1^, respectively). All amended treatments experienced a decrease in Cd accumulation by cassava roots to some extent, which could be attributed to dilution effects in root tissue and total plant biomass. Furthermore, the LCM treatment resulted in the greatest decrease in Cd accumulation after 3 months, which was approximately 5.6 times lower than that measured after the first month (*p* < 0.05). Higher GRDB values in this species were likely due to increased plant biomass, which decreased Cd concentration in roots. All soil amendments increased Cd accumulation by shoots and roots in jatropha and acacia by factors of up to 1.3 to 1.7 and 1.0 to 1.7, respectively. Cadmium levels in soil, on the other hand, have been demonstrated to increase with time as a result of increased sorption of Cd on OM^23^. Studies have also demonstrated that jatropha can serve as an accumulator for a variety of heavy metals; however, it is not a suitable accumulator for all heavy metals as it accumulates some at only very low quantities in shoots. Other reports claim that this species absorbs and accumulates substantial Cd in roots (3.2 to 8.6 mg kg^−1^). These data may indicate the effects of organic amendments on heavy metal stability in contaminated soil, as well as the excluder potential of jatropha^24^. In this study, jatropha shoots had roughly 1 to 2.6 times higher Cd concentrations compared to roots across all treatments. Plants in the SBM, SLVC, and BMBM treatments, on the other hand, showed somewhat greater Cd levels in roots rather than in shoots.

The influence of the organic amendments on Cd accumulation are associated with the physicochemical properties of soils. For example, addition of animal manure and biochar to soil reduced Cd mobility and enhanced Cd stabilization as a result of increased Cd precipitation and sorption via elevated soil pH and CEC. These effects resulted in decreased Cd bioavailability and translocation through plant tissues^25^. In the current study, leonardite had a low pH value (2.6); however, its OM content is substantial (20.1%). The OM will decrease Cd mobility because certain OM components produce ligands that bind and chelate heavy metal ions and form stable complexes^1^. Compared with other treatments, the SLBM treatment (acacia) exhibited remarkably low Cd concentrations in roots and stems (3.5 mg kg^−1^ and 2.9 mg kg^−1^, respectively); nevertheless, the greatest Cd concentration (4.2 mg kg^−1^) was found in leaves (*p* < 0.05).

The Cd uptake values of the study plants were in the order: jatropha > cassava > acacia (Fig. 3D). Jatropha took up substantially more Cd than any other plant species (*p* < 0.05). Acacia and cassava showed remarkably low Cd concentrations in the SLBM treatment (3.5 mg kg^−1^ and 3 mg kg^−1^, respectively), which followed a pattern similar to Cd uptake values (35 mg plant^−1^ and 119.1 mg plant^−1^, respectively). The BMCM treatment for jatropha showed substantial total and Ext. soil Cd concentrations, however, this treatment had a low Cd uptake value for jatropha (127.8 mg plant^−1^), implying that a certain soil amendment could stabilize and reduce phytoavailability of Cd.

Acacia in the SLVC treatment exhibited substantially elevated Cd concentrations in roots (5 mg kg^−1^; *p* < 0.05) although the treatment had a high OM content (9.9%). However, leonardite application reduced soil pH, as evidenced by the acidic character of the SLVC treatment (pH 5.5), which will result in increased Cd mobility in the soil and thus greater bioavailability. Despite the fact that BMVC had soil pH near neutral (pH 6.8) and high OM content (9.6%), the Cd concentration in acacia roots was greatest in the BMVC treatment (5.1 mg Cd kg^−1^; *p* < 0.05). To attain the low Cd concentrations in plant tissue found in the SLBM treatment, soil pH may need to be adjusted to a mildly alkaline level. However, the high OM content (8.6%) in this treatment might be a key factor in reducing Cd bioavailability to the plants. If Cd phytoextraction is the primary goal, however, the BMBM treatment may be the best option, as all study plants showed substantial Cd uptake values, i.e., 187.9, 175.1, and 123.9 mg plant^−1^ for jatropha, cassava and acacia, respectively.

Jatropha seeds are typically released in the field when trees reach a height of about 2 to 3 m; however, jatropha seeds were present in pots during this greenhouse study, even though plants did not reach these heights. Jatropha seeds were only found in the SBM and SLVC treatments. Seeds of jatropha are, incidentally, often reported to be toxic^26^. The bone meal in the SBM treatment constituted a suitable amendment for promoting growth until seed production.

The ability of cassava roots to produce and accumulate starch in agricultural areas in Thailand is dependent on growth period, which is often as long as 9 to 12 months. Cassava tubers were found in many pot treatments such as SBM, SLVC, LBM, BMVC, and BMCM, indicating that many amendments can promote growth and production of cassava. The tubers were, however, young and could not yet be consumed. Cadmium concentrations in those edible plant parts were similarly found to be low albeit higher than the CODEX Alimentarius Commission, Joint FAO/WHO Food Standard Program standard threshold (> 0.4 mg kg^−1^)^27^.

### Phytomanagement of Cd by the study plants

All plants examined under the range of treatments had TF values < 1, with values changing only slightly from first to third month of growth (*p* > 0.05) (Tables 2 and S1); these values indicate Cd retention in roots. This effect could be a result of characteristics unique to the excluder phenotype making it suitable for phytostabilization purposes^13^. Cassava exhibited 1.6 to 6.3 times higher BCFR values from the first to the third month of growth, and jatropha and acacia had lower BCFR values (Tables 2 and S1). BCFR values for all plants in all treatments were typically > 1, except for the BMVC treatment for jatropha (0.9). All BCFR values in all treatments for *M. esculenta* were notably high or in the range of 3.6–14.4 with TF values < 1, except for the LCM treatment (1.1). Furthermore, the SLVC treatments for acacia and jatropha had substantial BCFR values (2.2 and 2.0, respectively) and lowest TF values (0.6 and 0.7, respectively). BCFR values > 1 and TF values < 1 may reflect the phytostabilization potential of the study plants^13,14^. Jatropha in the LCM treatment had a low BCFR value (1.0) and a TF value greater than 1. Jatropha is a perennial bioenergy crop that can be used for phytoextraction^28^. This perennial plant cultivated in mine spoils mixed with peat moss translocated large quantities of metals from roots to aerial portions, which is a crucial characteristic of accumulator and hyperaccumulator plants^28^. Based on the results the current study, however, jatropha cannot be designated a hyperaccumulator of Cd because its TF values are generally < 1, while BCFR values are > 1. As such, their Cd accumulation levels did not meet the criteria of Baker and Brooks^29^, which require Cd levels in shoots to be > 100 mg kg^−1^. Based on the ability of Cd accumulation in roots or tubers by the study plants, cassava and acacia were classified as Cd excluders, indicating that they avoid importing metals to aerial parts. However, cassava peels may be the primary storage organ for heavy metals such as Pb^30^.

**Table 2:** Bioconcentration factor for root (BCFR) and translocation factor (TF) among the study plants after harvest (*n* = 4).

Scientific name	Treatment	BCFR	TF
*J. curcas*	SL	1.2	0.8
	SBM	1.2	0.7
	SLBM	1.3	0.7
	SLVC	2	0.7
	LCM	1	1.3
	LBM	1	0.9
	BMVC	0.9	0.8
	BMCM	1.3	0.7
	BMBM	1.2	0.9
*M. esculenta*	SL	10.2	0.8
	SBM	3.6	0.8
	SLBM	5.6	0.9
	SLVC	11.2	0.8
	LCM	8.5	1.1
	LBM	6.8	0.9
	BMVC	12.4	0.9
	BMCM	14.4	0.8
	BMBM	4.1	0.8
*A. mangium*	SL	3.8	0.7
	SBM	1.8	0.8
	SLBM	1.1	0.9
	SLVC	2.2	0.6
	LCM	1.7	0.6
	LBM	1.4	0.7
	BMVC	1.5	0.6
	BMCM	1.5	0.8
	BMBM	1.6	0.8

Plants suited for phytomanagement in heavy metal-contaminated locations may not have a significant capacity to accumulate high levels of heavy metals; however, they may offer benefits as a non-edible product. According to Abdelsalam et al.^6^, potential bioenergy crops or non-edible crops, for example, can uptake and accumulate heavy metals in various plant organs; these plant tissues and crude extracts regardless provide benefits for multiple purposes such as biofuel; and fiber, paper and wood production. Cassava is another appealing bioenergy crop for the reclamation of metal-polluted soils because this tuber crop is easy to cultivate in tropical and subtropical areas, and cassava tubers contain a high content of starch that could be used commercially as a renewable feedstock to produce ethanol fuel^31^. Jatropha has advantageous properties such as easy propagation, rapid growth, and drought tolerance, thus it can be grown widely in locations globally^32^. This species offers promise for sustainable industrial application as its components can be used as fertilizer, insecticide, soap, medicine, and energy source. Despite the fact that neither cassava nor jatropha are hyperaccumulators, they are non-food bioenergy crops that can be cultivated in harsh environments including heavy metal-contaminated sites^31^. Jatropha demonstrated a great capacity for phytoremediation of several metals (iron (Fe), Cr, manganese (Mn), and Cu) from fly ash in greenhouse studies—heavy metal uptake increased by 117% in roots, 62% in stems, and 86% in leaves, respectively, compared to control^31,33^. Jatropha and cassava, which may be grown in heavily Cd-contaminated soils and used for renewable energy or biodiesel, are considered suitable for long-term use and rehabilitation of heavy metal-contaminated lands^31,34^.

The growth data for acacia compared with that of the other study plants revealed that this perennial species had the highest dry biomass and GRDB, as well as low Cd accumulation and no toxicity symptoms. Acacia, an N-fixing plant, can fix atmospheric N_2_ via symbiotic root nodules and does not pose any major invasive concerns^35^. Acacia wood is heavy, hard, robust and durable; thus, it is well-suited for furniture and structural purposes^36^. Perennial bioenergy crops require lower nutrient inputs and have higher lignin and cellulose content than biomass from annual crops^37^. Therefore, acacia could be cultivated over large tracts of Cd-contaminated land to produce extensive biomass, making it a suitable candidate as a plant-based energy source.

## Conclusions

Plant biomass occurring in contaminated sites often accumulates high quantities of heavy metals, rendering it inappropriate for either human consumption or animal fodder. Contaminated plant biomass can, however, be anaerobically digested or combusted for energy production. Bioenergy crops can replace edible crops in heavy metal-contaminated soils, allowing for long-term use and rehabilitation and providing substantial benefits to local communities and farmers. Perennial plants, *e.g.,* acacia and jatropha have low cultivation requirements and are a cost-effective long-term option. Furthermore, the optimal species for phytomanagement must have a high BCFR and low TF values. Jatropha may be the most suitable species, as evidenced by having the greatest BCFR and the lowest TF values in the BMCM treatment. Following harvest, BMCM-treated soil had slightly higher Cd concentrations than several other treatments (3.1 mg kg^−1^) but low Cd accumulation and uptake values were observed, indicating that this amendment limited Cd bioavailability. The bone meal, leonardite, and bat manure were also beneficial in improving plant growth and development. Substantial growth performance of jatropha and acacia without Cd toxicity, and marked Cd accumulation in roots indicate their potential for cultivation of former zinc mine sites (or locations downriver) exposed to high Cd concentrations.

## Supplementary Information

Supplementary Tables.

## Data Availability

The data that support the findings of this study are available on request from the corresponding author.
