# Supplementary material for: Organic amendment additions to cadmium-contaminated soils for phytostabilization of three bioenergy crops
Source: Sci Rep. 2022 Jul 29;12:13070. doi: 10.1038/s41598-022-17385-8 (PMC9338034; doi:10.1038/s41598-022-17385-8)
Supplement: Supplementary file 1 — Supplementary Tables. [file 41598_2022_17385_MOESM1_ESM.docx]

**Table S1** Growth performance of study plants, for 3 months (*n* = 4)

| Scientific name | Treatment | Month | Height (cm) | Root (cm) | Biomass (g plant^-1^) | Growth rate in dry biomass |
| --- | --- | --- | --- | --- | --- | --- |
|  |  |  |  |  |  |  |
| *J. curcas* | SL | 0 | 36.7±0.6aA^2^ | 0.0±0.0aB^3^ | 11.0±1.7aA^3^ | - |
|  |  | 1 | 51.3±2.1aB^1^ | 14.5±1.1aC^2^ | 27.8±2.3aA^2^ | 17.4±2.3aA^1^ |
|  |  | 3 | 50.8±5.0bA^1^ | 20.3±2.7bC^1^ | 59.9±7.4abA^1^ | 16.3±2.5abA^1^ |
|  | SBM | 0 | 36.7±0.6aA^2^ | 0.0±0.0aB^3^ | 11.0±1.7aA^3^ | - |
|  |  | 1 | 43.7±4.7abcB^2^ | 11.4±5.1abB^2^ | 33.0±5.9aA^2^ | 22.5±5.9aA^1^ |
|  |  | 3 | 66.1±10.5aA^1^ | 24.5±4.1abA^1^ | 62.5±2.5aA^1^ | 17.2±0.8aA^1^ |
|  | SLBM | 0 | 36.7±0.6aA^2^ | 0.0±0.0aB^3^ | 11.0±1.7aA^3^ | - |
|  |  | 1 | 38.8±4.3cB^2^ | 12.1±6.6abB^2^ | 25.7±5.6aA^2^ | 15.3±5.6aA^1^ |
|  |  | 3 | 60.8±2.3abA^1^ | 29.6±11.3aA^1^ | 52.4±8.7abcA^1^ | 13.8±2.9abcA^1^ |
|  | SLVC | 0 | 36.7±0.6aA^2^ | 0.0±0.0aB^2^ | 11.0±1.7aA^2^ | - |
|  |  | 1 | 47.6±11.7abB^1,2^ | 10.6±5.7abB^1^ | 27.2±12.4aA^1,2^ | 16.8±12.4aA^1^ |
|  |  | 3 | 55.2±13.4abA^1^ | 19.3±1.7bB^1^ | 42.4±16.0bcB^1^ | 10.5±5.3bcB^1^ |
|  | LCM | 0 | 36.7±0.6aA^2^ | 0.0±0.0aB^3^ | 11.0±1.7aA^2^ | - |
|  |  | 1 | 38.2±6.7cA^2^ | 7.1±1.6bcB^2^ | 14.3±4.0bA^2^ | 3.8±4.0bA^1^ |
|  |  | 3 | 58.9±13.6abA^1^ | 16.3±5.6bB^1^ | 46.8±21.1abcA^1^ | 11.9±7.0abcA^1^ |
|  | LBM | 0 | 36.7±0.6aA^3^ | 0.0±0.0aB^3^ | 11.0±1.7aA^3^ | - |
|  |  | 1 | 45.3±1.9abcB^2^ | 11.0±1.7abB^2^ | 25.6±3.6aA^2^ | 15.1±3.6aA^1^ |
|  |  | 3 | 58.0±8.9abB^1^ | 22.5±1.7abAB^1^ | 57.4±13.3abA^1^ | 15.5±4.4abA^1^ |
|  | BMVC | 0 | 36.7±0.6aA^1^ | 0.0±0.0aB^3^ | 11.0±1.7aA^2^ | - |
|  |  | 1 | 41.7±3.3bcB^1^ | 6.2±5.0bcB^2^ | 28.9±7.6aA^1^ | 18.6±7.7aA^1^ |
|  |  | 3 | 50.3±14.8bA^1^ | 20.5±5.9bA^1^ | 42.1±15.0bcA^1^ | 10.4±5.0bcA^1^ |
|  | BMCM | 0 | 36.7±0.6aA^2^ | 0.0±0.0aB^3^ | 11.0±1.7aA^3^ | - |
|  |  | 1 | 38.4±3.0 cA^2^ | 3.5±1.8cB^2^ | 23.1±7.2abA^2^ | 12.7±7.2abA^1^ |
|  |  | 3 | 53.1±12.4abAB^1^ | 24.1±3.0abA^1^ | 39.0±10.0cAB^1^ | 9.4±3.3cA^1^ |
|  | BMBM | 0 | 36.7±0.6aA^3^ | 0.0±0.0aB^3^ | 11.0±1.7aA^3^ | - |
|  |  | 1 | 47.0±2.7abA^2^ | 11.9±5.1abB^2^ | 23.6±9.6abA^2^ | 13.1±9.6abA^1^ |
|  |  | 3 | 61.8±1.3abA^1^ | 23.6±2.5abA^1^ | 53.3±9.6abcB^1^ | 14.1±3.2abcB^1^ |
| *M. esculenta* | SL | 0 | 35.7±0.7aA^2^ | 0.0±0.0aB^2^ | 8.3±1.4aB^2^ | - |
|  |  | 1 | 61.1±5.0aA^1^ | 22.8±3.2abB^1^ | 19.5±7.4aB^2^ | 11.2±7.4aAB^1^ |
|  |  | 3 | 60.8±1.9bA^1^ | 27.3±5.1abcdB^1^ | 56.7±17.8aA^1^ | 16.1±5.9aA^1^ |
|  | SBM | 0 | 35.7±0.7aA^2^ | 0.0±0.0aB^3^ | 8.3±1.4aB^2^ | - |
|  |  | 1 | 62.0±7.2aA^1^ | 17.0±8.7abcAB^2^ | 21.7±3.5aB^2^ | 13.4±3.5aB^1^ |
|  |  | 3 | 66.5±8.7abA^1^ | 33.8±6.3abA^1^ | 40.7±22.7aA^1^ | 14.9±7.5aA^1^ |
|  | SLBM | 0 | 35.7±0.7aA^3^ | 0.0±0.0aB^3^ | 8.3±1.4aB^2^ | - |
|  |  | 1 | 58.1±3.5abA^2^ | 13.6±6.3cB^2^ | 21.1±8.48aAB^2^ | 12.8±8.5aA^1^ |
|  |  | 3 | 67.5±5.1abA^1^ | 36.3±13.2aA^1^ | 51.0±22.9aA^1^ | 14.2±7.6aA^1^ |
|  | SLVC | 0 | 35.7±0.7aA^2^ | 0.0±0.0aB^2^ | 8.3±1.4aB^2^ | - |
|  |  | 1 | 65.2±8.2aA^1^ | 22.9±1.9abA^1^ | 15.0±5.9aAB^2^ | 6.7±5.9aA^2^ |
|  |  | 3 | 71.8±2.6aA^1^ | 28.1±6.4abcdAB^1^ | 48.0±15.8aA^1^ | 18.8±4.5aA^1^ |
|  | LCM | 0 | 35.7±0.7aA^3^ | 0.0±0.0aB^2^ | 8.3±1.4aB^2^ | - |
|  |  | 1 | 43.9±6.3cA^2^ | 15.1±8.1bcB^1^ | 13.0±4.8aA^2^ | 4.6±4.8aA^2^ |
|  |  | 3 | 70.3±5.3aA^1^ | 17.6±1.5dB^1^ | 64.4±7.5aA^1^ | 18.7±2.5aA^1^ |
|  | LBM | 0 | 35.7±0.7aA^3^ | 0.0±0.0aB^2^ | 8.3±1.4aB^2^ | - |
|  |  | 1 | 64.9±4.6aA^2^ | 25.0±6.3aA^1^ | 19.4±8.4aAB^2^ | 11.1±8.4aA^1^ |
|  |  | 3 | 73.1±4.9a A ^1^ | 19.8±4.1cdB^1^ | 75.1±41.6aA^1^ | 22.2±13.9aA^1^ |
|  | BMVC | 0 | 35.7±0.7aA^2^ | 0.0±0.0aB^2^ | 8.3±1.4aB^2^ | - |
|  |  | 1 | 58.8±1.7abA^1^ | 20.3±9.3abcA^1^ | 15.8±4.6aB^2^ | 7.5±4.6aB^2^ |
|  |  | 3 | 64.1±8.8abA^1^ | 29.4±10.6abcdA^1^ | 41.9±7.3aA^1^ | 16.0±3.9aA^1^ |
|  | BMCM | 0 | 35.7±0.7aA^3^ | 0.0±0.0aB^2^ | 8.3±1.4aB^2^ | - |
|  |  | 1 | 50.2±11.4bcA^2^ | 14.5±3.1bcA^1^ | 17.5±7.0aA^2^ | 9.2±7.0aA^1^ |
|  |  | 3 | 65.0±8.8abA^1^ | 21.9±4.3bcdA^1^ | 36.6±17.0aA^1^ | 17.3±9.9aA^1^ |
|  | BMBM | 0 | 35.7±0.7aA^3^ | 0.0±0.0aB^2^ | 8.3±1.4aB^2^ | - |
|  |  | 1 | 45.3±8.4cA^2^ | 13.4±2.1cB^1^ | 15.2±3.3aA^2^ | 6.9±3.3aA^2^ |
|  |  | 3 | 61.1±5.5bA^1^ | 31.5±19.2abcA^1^ | 45.0±18.8aA^1^ | 22.5±3.9aA^1^ |
| *A. mangium* | SL | 0 | 17.4±1.7aB^3^ | 12.9±3.0aA^2^ | 3.6±0.4aC^2^ | - |
|  |  | 1 | 28.3±4.8 abcdC^2^ | 34.5±6.0aA^1^ | 9.6±3.4bC^2^ | 6.1±3.4bB^2^ |
|  |  | 3 | 56.7±10.3 abcdA^1^ | 40.8±4.1aA^1^ | 17.8±6.8bcdB^1^ | 14.3±6.8bcdA^1^ |
|  | SBM | 0 | 17.4±1.7aB^3^ | 12.9±3.0aA^2^ | 3.6±0.4aC^2^ | - |
|  |  | 1 | 34.5±6.0 abB^2^ | 25.8±7.6abA^1^ | 10.7±2.6bC^2^ | 7.1±2.6bB^2^ |
|  |  | 3 | 68.9±12.9 abA^1^ | 29.8±7.8abcA^1^ | 23.0±9.0abB^1^ | 19.4±9.0abA^1^ |
|  | SLBM | 0 | 17.4±1.7aB^2^ | 12.9±3.0aA^2^ | 3.6±0.4aC^2^ | - |
|  |  | 1 | 37.1±12.2aB^2^ | 29.2±11.1abA^1^ | 14.0±4.8abB^1^ | 10.5±4.9abA^1^ |
|  |  | 3 | 74.1±26.3aA^1^ | 33.0±11.6abcA^1^ | 9.6±5.36cdB^1,2^ | 6.1±5.4cdA^1,2^ |
|  | SLVC | 0 | 17.4±1.7aB^2^ | 12.9±3.0aA^2^ | 3.6±0.4aC^2^ | - |
|  |  | 1 | 28.9±5.9abcdC^2^ | 31.6±8.9abcdA^1^ | 10.8±4.0bB^1^ | 7.3±4.0bA^1^ |
|  |  | 3 | 57.8±12.8abcdA^1^ | 36.6±7.0abA^1^ | 8.6±3.7dC^1^ | 5.1±3.7dB^1^ |
|  | LCM | 0 | 17.4±1.7aB^2^ | 12.9±3.0aA^2^ | 3.6±0.4aC^1^ | - |
|  |  | 1 | 18.6±1.4dB^2^ | 26.6±6.0dA^1^ | 10.4±3.0bA^1^ | 6.9±3.0bA^1^ |
|  |  | 3 | 37.1±3.0dB^1^ | 32.6±6.1abcA^1^ | 10.9±9.4cdB^1^ | 9.8±9.7bcdA^1^ |
|  | LBM | 0 | 17.4±1.7aB^2^ | 12.9±3.0aA^2^ | 3.6±0.4aC^2^ | - |
|  |  | 1 | 20.7±3.8cdC^2^ | 22.6±8.3cdA^1^ | 13.0±1.9abB^1^ | 9.4±1.9abA^1^ |
|  |  | 3 | 27.5±3.8cdC^1^ | 41.4±8.2bcA^1^ | 11.3±6.2cdB^1^ | 7.7±6.2cdA^1^ |
|  | BMVC | 0 | 17.4±1.7aB^2^ | 12.9±3.0aA^2^ | 3.6±0.4aC^2^ | - |
|  |  | 1 | 30.6±9.8abcdC^2^ | 25.8±6.3abcdA^1^ | 14.1±2.2abB^1^ | 10.5±2.2abAB^1,2^ |
|  |  | 3 | 61.1±21.3abcdA^1^ | 30.8±8.9abcA^1^ | 18.7±7.6abcB^1^ | 15.2±7.6abcA^1^ |
|  | BMCM | 0 | 17.4±1.7aB^2^ | 12.9±3.0aA^2^ | 3.6±0.4aC^2^ | - |
|  |  | 1 | 22.6±4.1bcdB^2^ | 21.3±5.6bcdA^1^ | 15.6±2.1aA^2^ | 12.0±2.1aA^2^ |
|  |  | 3 | 45.3±8.9bcdB^1^ | 22.0±5.9cA^1^ | 21.7±5.1aA^2^ | 18.2±5.1abA^1^ |
|  | BMBM | 0 | 17.4±1.7aB^2^ | 12.9±3.0aA^2^ | 3.6±0.4aC^1^ | - |
|  |  | 1 | 32.3±17.8abcA^1,2^ | 24.9±8.0abcA^1,2^ | 15.8±2.5aA^2^ | 12.3±2.5aA^1^ |
|  |  | 3 | 64.6±38.4abcA^1^ | 35.1±12.3abA^1^ | 27.7±5.9aC^1^ | 24.2±5.9aA^1^ |

Values followed by the same letter are not significantly different; lower-case letters show the differences in growth performance among treatments within the same plant species and growth period (LSD, *p* < 0.05); capital letters show the differences in growth performance among plant species within the same treatment (LSD: *p* < 0.05); numbers indicate the differences in growth performance among growth periods within the same plant species and treatment.

**Table S2** Cd accumulation and uptake, bioconcentration factor for root (BCFR) and translocation factor (TF) among study plants, 3 months (*n* = 4)

| Scientific name | Treatment | Month | Cd accumulation in plants (mg kg^-1^) | | | | | | Cd uptake (mg plant^-1^) | BCFR | TF |
| --- | --- | --- | --- | --- | --- | --- | --- | --- | --- | --- | --- |
|  |  |  | **Leaf** | **Stem** | **Root** | **Cassava tuber** | **Seed** | **Whole plant** |  |  |  |
| *J. curcas* | SL | 0 | BDL | BDL | BDL | - | - | BDL | BDL | - | - |
|  |  | 1 | 3.3±1.0aA^2^ | 3.4±1.2aA^1^ | 5.9±1.5aA^1^ | - | - | 4.2±0.6aA^1^ | 120.1±20.4aA^2^ | 6.3±1.2abA^1^ | 0.6±0.1cdA^1^ |
|  |  | 3 | 4.4±0.8bA^1^ | 2.3±0.8bcB^2^ | 3.2±2.2abA^2^ | - | - | 3.3±0.8aAB^2^ | 197.0±22.8abA^1^ | 1.2±0.3abB^2^ | 0.8±0.3cdA^1^ |
|  | SBM | 0 | BDL | BDL | BDL | - | - | BDL | BDL | - | - |
|  |  | 1 | 3.3±1.0aA^2^ | 3.1±1.2abA^1^ | 4.0±1.1cA^1^ | - | - | 3.5±0.5bcA^1^ | 116.2±21.7abA^2^ | 3.6±0.8abcA^1^ | 0.8±0.3bAB^1^ |
|  |  | 3 | 4.4±0.8bA^1^ | 2.5±0.7abcA^2^ | 3.5±1.6abA^1^ | - | 0.6±0.3a | 3.5±0.6aA^1^ | 217.0±13.1aA^1^ | 1.2±0.1abcB^2^ | 0.7±0.1bA^1^ |
|  | SLBM | 0 | BDL | BDL | BDL | - | - | BDL | BDL | - | - |
|  |  | 1 | 2.8±1.0aA^2^ | 2.9±1.1bA^1^ | 3.9±1.9cAB^1^ | - | - | 3.0±0.8cdAB^2^ | 79.1±17.7abcA^2^ | 9.1±2.3abcA^1^ | 0.8±0.2bcA^1^ |
|  |  | 3 | 4.8±1.0abA^1^ | 2.2±0.7cB^2^ | 3.2±1.7abA^1^ | - | - | 3.4±0.8aA^1^ | 177.8±32.4abA^1^ | 1.3±0.3abcB^1^ | 0.7±0.1bcB^1^ |
|  | SLVC | 0 | BDL | BDL | BDL | - | - | BDL | BDL | - | - |
|  |  | 1 | 2.9±1.1aA^2^ | 2.9±1.1bA^1^ | 6.0±1.3aA^1^ | - | - | 3.6±0.9abcA^1^ | 102.4±53.3abA^1^ | 11.3±6.2aA^1^ | 0.5±0.0dB^2^ |
|  |  | 3 | 4.2±0.8bA^1^ | 2.6±0.9abcA^1^ | 3.9±1.9aB^2^ | - | 0.5±0.1a | 3.3±1.0aAB^1^ | 147.6±74.5abA^1^ | 2.0±0.3aB^1^ | 0.7±0.1dAB^1^ |
|  | LCM | 0 | BDL | BDL | BDL | - | - | BDL | BDL | - | - |
|  |  | 1 | 3.3±1.3aA^1^ | 3.2±0.8abA^1^ | 5.0±1.0bB^1^ | - | - | 3.4±0.8abA^1^ | 54.8±12.0cAB^2^ | 3.8±1.6bcA^1^ | 0.6±0.0bcdB^1^ |
|  |  | 3 | 4.4±1.0bA^1^ | 2.6±0.8abcA^1^ | 2.2±1.7bB^2^ | - | - | 3.0±0.9aA^1^ | 145.2±68.8abA^1^ | 1.0±0.5bcB^1^ | 1.3±0.8bcdA^1^ |
|  | LBM | 0 | BDL | BDL | BDL | - | - | BDL | BDL | - | - |
|  |  | 1 | 3.0±1.0aA^2^ | 2.9±1.2bA^1^ | 2.4±0.5dB^1^ | - | - | 2.7±0.4dB^2^ | 71.3±11.3bcA^2^ | 1.3±0.5cA^1^ | 1.2±0.1aA^1^ |
|  |  | 3 | 4.6±0.8abA^1^ | 2.5±0.9abcA^1^ | 2.9±1.7abB^1^ | - | - | 3.4±0.5aA^1^ | 193.3±46.8abA^1^ | 1.0±0.3cB^1^ | 0.9±0.2aA^2^ |
|  | BMVC | 0 | BDL | BDL | BDL | - | - | BDL | BDL | - | - |
|  |  | 1 | 3.2±1.1aA^2^ | 2.9±0.8bA^1^ | 5.1±0.9bA^1^ | - | - | 3.2±0.8abA^1^ | 109.5±24.1abA^1^ | 4.8±0.8abcA^1^ | 0.6±0.0cdB^2^ |
|  |  | 3 | 5.1±1.2aA^1^ | 2.7±0.7abA^1^ | 3.4±1.7abB^2^ | - | - | 3.5±0.8aAB^1^ | 151.9±68.4abA^1^ | 0.9±0.1abcB^2^ | 0.8±0.1cdA^1^ |
|  | BMCM | 0 | BDL | BDL | BDL | - | - | BDL | BDL | - | - |
|  |  | 1 | 3.1±0.9aA^2^ | 3.1±1.3abA^1^ | 6.2±1.8aA^1^ | - | - | 3.5±1.3aA^1^ | 101.4±47.4abA^1^ | 7.5±1.1abcA^1^ | 0.5±0.1aB^1^ |
|  |  | 3 | 4.5±0.8abA^1^ | 2.7±0.9abcA^1^ | 2.9±2.1abA^2^ | - | - | 3.2±1.0aA^1^ | 127.8±60.3bA^1^ | 1.3±0.2abcB^2^ | 0.7±0.1dA^1^ |
|  | BMBM | 0 | BDL | BDL | BDL | - | - | BDL | BDL | - | - |
|  |  | 1 | 2.9±1.0aA^2^ | 3.0±1.0abA^1^ | 4.9±1.2bB^2^ | - | - | 3.4±0.8bcA^1^ | 83.8±41.9abcA^2^ | 6.3±2.1abcA^1^ | 0.6±0.1cdA^2^ |
|  |  | 3 | 4.6±0.9abA^1^ | 2.9±0.8aAB^1^ | 3.1±1.6abB^2^ | - | - | 3.5±0.7aA^1^ | 187.9±32.9abA^1^ | 1.2±0.3abcA^1^ | 0.9±0.1cdA^1^ |
| *M. esculenta* | SL | 0 | BDL | BDL | BDL | - | - | BDL | BDL | - | - |
|  |  | 1 | 2.5±0.9abB^1^ | 1.8±1.1dC^2^ | 5.7±2.4bA^1^ | - | - | 3.3±0.6bB^1^ | 53.6±18.2aB^2^ | 2.2±0.4abB^2^ | 0.3±0.0cB^2^ |
|  |  | 3 | 1.8±0.5cdC^2^ | 3.1±0.5aA^1^ | 3.9±1.4abA^2^ | - | - | 2.9±0.6bcB^1^ | 154.7±51.7aA^1^ | 10.2±3.5abcdA^1^ | 0.8±0.0bA^1^ |
|  | SBM | 0 | BDL | BDL | BDL | - | - | BDL | BDL | - | - |
|  |  | 1 | 2.8±0.6aA^1^ | 2.3±1.1abcB^1^ | 4.0±1.8bA^1^ | - | - | 3.0±0.4bA^1^ | 72.4±20.1aB^1^ | 1.5±0.3bB^2^ | 0.6±0.2aB^1^ |
|  |  | 3 | 2.5±0.5aB^1^ | 2.8±0.7aA^1^ | 3.4±1.6bA^1^ | 1.6±0.3b | - | 2.9±0.6bcdB^1^ | 126.9±41.4aB^1^ | 3.6±0.6dA^1^ | 0.8±0.2bA^1^ |
|  | SLBM | 0 | BDL | BDL | BDL | - | - | BDL | BDL | - | - |
|  |  | 1 | 2.7±0.9aA^1^ | 2.8±0.9aA^1^ | 4.2±1.4bA^1^ | - | - | 3.2±0.3bA^1^ | 68.4±29.2aA^1^ | 1.2±0.3bA^2^ | 0.7±0.1aA^2^ |
|  |  | 3 | 2.4±0.5aC^1^ | 3.2±0.4aA^1^ | 3.4±1.7abA^2^ | - | - | 3.0±0.7abB^1^ | 119.1±62.0aA^1^ | 5.6±1.5bcdA^1^ | 0.9±0.1abA^1^ |
|  | SLVC | 0 | BDL | BDL | BDL | - | - | BDL | BDL | - | - |
|  |  | 1 | 2.5±1.1abA^1^ | 2.6±1.1abcA^1^ | 4.6±1.3bB^1^ | - | - | 3.2±0.3bAB^1^ | 46.1±16.2aB^2^ | 1.9±0.2bA^2^ | 0.6±0.1aAB^2^ |
|  |  | 3 | 1.9±0.6bcdB^2^ | 3.0±0.6aA^1^ | 3.8±1.6abB^1^ | 6.0±4.9ab | - | 2.9±0.6bcdB^1^ | 152.6±40.4aA^1^ | 11.2±5.2abA^1^ | 0.8±0.1bA^1^ |
|  | LCM | 0 | BDL | BDL | BDL | - | - | BDL | BDL | - | - |
|  |  | 1 | 1.9±1.2bB^1^ | 2.6±0.9abB^1^ | 15.0±10.2aA^1^ | - | - | 5.4±3.5aA^1^ | 67.1±36.8aA^2^ | 6.4±2.5aA^1^ | 0.2±0.1cC^2^ |
|  |  | 3 | 2.3±0.6abB^1^ | 2.9±0.5aA^1^ | 2.7±1.6cB^2^ | - | - | 2.6±0.6cdA^1^ | 132.5±30.0aAB^1^ | 8.5±3.4abcdA^1^ | 1.1±0.2aA^1^ |
|  | LBM | 0 | BDL | BDL | BDL | - | - | BDL | BDL | - | - |
|  |  | 1 | 2.7±0.7aAB^1^ | 2.3±1.2bcdB^1^ | 4.6±1.5bA^1^ | - | - | 3.2±0.3 bA^1^ | 62.2±26.4aA^1^ | 1.4±0.3bA^2^ | 0.5±0.0abC^1^ |
|  |  | 3 | 1.6±0.5dC^2^ | 2.8±0.6aA^1^ | 3.3±1.6bcB^2^ | 5.1±1.1bc | - | 2.6±0.5dB^2^ | 154.9±86.6aA^1^ | 6.8±1.3bcdA^1^ | 0.9±0.4abA^1^ |
|  | BMVC | 0 | BDL | BDL | BDL | - | - | BDL | BDL | - | - |
|  |  | 1 | 2.5±1.1aA^1^ | 2.5±1.0abcA^1^ | 4.5±1.3bB^1^ | - | - | 3.2±0.4bB^1^ | 51.9±17.0aB^2^ | 1.5±0.3bB^1^ | 0.6±0.1aB^2^ |
|  |  | 3 | 2.3±0.5abB^1^ | 2.9±0.6aA^1^ | 3.3±1.5bcB^2^ | 4.9±0.4bc | - | 2.8±0.6bcdB^2^ | 129.8±26.1aA^1^ | 12.4±8.6abcA^1^ | 0.9±0.1abA^1^ |
|  | BMCM | 0 | BDL | BDL | BDL | - | - | BDL | BDL | - | - |
|  |  | 1 | 2.7±0.8 aA^1^ | 2.4±1.0abcB^2^ | 6.5±3.0bA^2^ | - | - | 3.9±0.8abAB^1^ | 64.9±18.0aA^2^ | 2.3±0.7abA^2^ | 0.4±0.1bcB^2^ |
|  |  | 3 | 2.5±0.5 aB^1^ | 3.3±0.7aA^1^ | 4.1±1.8aA^1^ | 7.5±0.6a | - | 3.3±0.7aA^1^ | 145.5±58.9aA^1^ | 14.4±6.6aA^1^ | 0.8±0.1bA^1^ |
|  | BMBM | 0 | BDL | BDL | BDL | - | - | BDL | BDL | - | - |
|  |  | 1 | 2.3±1.0abA^1^ | 2.1±1.3cdB^1^ | 4.2±1.6bA^1^ | - | - | 2.9±0.4bB^1^ | 40.0±12.8aB^2^ | 1.5±0.3bA^2^ | 0.5±0.2abA^2^ |
|  |  | 3 | 2.1±0.4abcB^1^ | 2.7±0.4aB^1^ | 3.5±1.4abB^2^ | - | - | 2.8±0.6bcdB^1^ | 175.1±28.5aA^1^ | 4.1±1.2cdA^1^ | 0.8±0.1bA^1^ |
| *A. mangium* | SL | 0 | BDL | BDL | BDL | - | - | BDL | BDL | - | - |
|  |  | 1 | 2.8±0.3abcAB^1^ | 2.7±0.6abB^1^ | 3.6±0.4aB^1^ | - | - | 3.0±0.2abB^2^ | 33.4±8.0bcB^1^ | 3.1±0.5cdB^1^ | 0.8±0.2bA^1^ |
|  |  | 3 | 2.6±0.7bB^1^ | 3.1±0.2abA^1^ | 4.5±0.9abA^1^ | - | - | 3.4±0.2bA^1^ | 81.0±38.5abcB^1^ | 3.8±2.2aB^1^ | 0.7±0.1abcA^1^ |
|  | SBM | 0 | BDL | BDL | BDL | - | - | BDL | BDL | - | - |
|  |  | 1 | 3.2±0.3abA^1^ | 3.0±0.3aA^1^ | 3.3±0.1abA^2^ | - | - | 3.2±0.2aA^2^ | 35.1±9.7bcC^2^ | 1.9±0.2dB^1^ | 0.9±0.1bA^1^ |
|  |  | 3 | 3.9±0.5abA^1^ | 3.1±0.3abA^1^ | 4.0±0.2bcA^1^ | - | - | 3.7±0.1abA^1^ | 91.2±35.6abB^1^ | 1.8±0.7bB^1^ | 0.8±0.1abA^1^ |
|  | SLBM | 0 | BDL | BDL | BDL | - | - | BDL | BDL | - | - |
|  |  | 1 | 2.7±0.3bcA^2^ | 2.2±1.2bA^1^ | 3.4±0.4abB^1^ | - | - | 2.8±0.3bB^2^ | 46.4±15.7abA^1^ | 2.0±0.6cdA^1^ | 0.7±0.4bA^1^ |
|  |  | 3 | 4.2±0.3aB^1^ | 2.9±0.4abA^1^ | 3.5±0.5cA^1^ | - | - | 3.5±0.2abA^1^ | 35.0±20.0cB^1^ | 1.1±0.3abB^1^ | 0.9±0.2aAB^1^ |
|  | SLVC | 0 | BDL | BDL | BDL | - | - | BDL | BDL | - | - |
|  |  | 1 | 2.4±0.5cA^1^ | 2.6±0.3abA^1^ | 4.0±0.1aC^2^ | - | - | 3.0±0.2abB^2^ | 43.2±16.2abcB^1^ | 5.9±2.2abA^1^ | 0.7±0.1bA^1^ |
|  |  | 3 | 3.2±0.9abA^1^ | 3.0±0.2abA^1^ | 5.0±0.7aA^1^ | - | - | 3.8±0.2aA^1^ | 41.7±13.5cB^1^ | 2.2±0.4abB^2^ | 0.6±0.1bcB^1^ |
|  | LCM | 0 | BDL | BDL | BDL | - | - | BDL | BDL | - | - |
|  |  | 1 | 3.4±0.4aA^1^ | 3.3±0.4aA^1^ | 2.7±0.7bB^2^ | - | - | 3.1±0.4abA^1^ | 27.1±6.9cB^1^ | 2.0±0.6cdA^1^ | 1.3±0.2aA^1^ |
|  |  | 3 | 3.5±0.8abA^1^ | 2.6±0.5bA^1^ | 4.7±1.1abA^1^ | - | - | 3.6±0.5abA^1^ | 54.1±52.8bcB^1^ | 1.7±0.6bB^1^ | 0.6±0.3bcA^2^ |
|  | LBM | 0 | BDL | BDL | BDL | - | - | BDL | BDL | - | - |
|  |  | 1 | 2.4±0.5cB^1^ | 2.7±0.2abA^1^ | 3.3±1.0abB^1^ | - | - | 2.8±0.2abB^2^ | 44.2±16.2abcA^1^ | 3.0±1.9cdA^1^ | 0.9±0.3bB^2^ |
|  |  | 3 | 3.0±1.4abB^1^ | 2.9±0.3abA^1^ | 4.5±0.2abA^1^ | - | - | 3.4±0.3bA^1^ | 49.9±27.1bcB^1^ | 1.4±0.0bB^1^ | 0.7±0.1abcA^1^ |
|  | BMVC | 0 | BDL | BDL | BDL | - | - | BDL | BDL | - | - |
|  |  | 1 | 3.1±0.7abA^1^ | 2.8±0.2abA^1^ | 3.4±0.4aC^2^ | - | - | 3.1±0.3abB^2^ | 47.8±8.3abB^2^ | 4.1±1.5bcA^1^ | 0.8±0.1bA^1^ |
|  |  | 3 | 3.9±1.0abA^1^ | 2.9±0.1bA^1^ | 5.1±0.1aA^1^ | - | - | 4.0±0.4aA^1^ | 94.0±34.7abA^1^ | 1.5±0.1bB^2^ | 0.6±0.0cB^2^ |
|  | BMCM | 0 | BDL | BDL | BDL | - | - | BDL | BDL | - | - |
|  |  | 1 | 2.6±0.3bcA^1^ | 2.7±0.3abAB^1^ | 3.6±0.4aB^2^ | - | - | 3.0±0.3abB^2^ | 56.2±12.0aA^2^ | 6.4±2.5aA^1^ | 0.8±0.1bA^1^ |
|  |  | 3 | 3.2±0.8abB^1^ | 3.2±0.4abA^1^ | 4.2±0.3bcA^1^ | - | - | 3.5±0.1abA^1^ | 91.1±24.1abA^1^ | 1.5±0.2bB^2^ | 0.8±0.1abcA^1^ |
|  | BMBM | 0 | BDL | BDL | BDL | - | - | BDL | BDL | - | - |
|  |  | 1 | 2.7±0.3bcA^1^ | 3.1±0.1aA^1^ | 3.4±0.4aB^2^ | - | - | 3.0±0.1abAB^2^ | 53.8±9.1aAB^2^ | 3.8±0.6cdA^1^ | 0.9±0.1bA^1^ |
|  |  | 3 | 3.0±1.0abB^1^ | 3.5±0.5aA^1^ | 4.5±0.3abA^1^ | - | - | 3.6±0.4abA^1^ | 123.9±23.0aB^1^ | 1.6±0.1bB^2^ | 0.8±0.1abcA^1^ |

Values followed by the same letter are not significantly different; lower-case letters show the differences in accumulation and translocation values among treatments within the same plant species and growth period (LSD, *p* < 0.05); capital letters show the differences in accumulation and translocation values among plant species within the same treatment (LSD: *p* < 0.05); numbers show the differences in accumulation and translocation values among growth periods within the same plant and treatment.
